# What healthcare leadership can do in a climate crisis

**DOI:** 10.1177/08404704231157035

**Published:** 2023-03-23

**Authors:** Myles Sergeant, Ana Hategan

**Affiliations:** 1 3710 McMaster University, Hamilton, Ontario, Canada.

## Abstract

Healthcare governing boards, executives, medical staff, health professionals, and allied staff members should all play a role in devising, promoting, and implementing solutions for climate change mitigation, which must extend beyond the boundaries of their own workplaces and healthcare institutions. Such actions can potentially influence not only healthcare professionals and their patients but also healthcare supply chains and entire communities. Thus, leaders of healthcare organizations can play a vital role in leading by example. The authors herein propose some initiatives for promoting and implementing a culture of sustainability and climate action in medicine.

## Why climate change is an important issue: Impact on people

There are only about seven years left until reaching the deadline for the goal of limiting global warming to 1.5°C above the pre-industrial levels.^1^ Scientists argue that it is “now or never” to limit global warming, with warnings that the world may be on a constant acceleration toward a catastrophe.^1^ According to the United Nations’ leaders, unless governments do something about this now, life on our planet will become unsustainable.^1^ To mitigate this adversity, we believe that climate action is required from all sectors of the economy, including healthcare. In Canada, this effort is crucial to achieve the net-zero carbon emission goal by 2050, while transitioning to climate-resilient operations that reduce environmental impacts beyond carbon, including on waste, water, and biodiversity.^2^ Therefore, it is essential that health leaders respond to climate change in their own institutions.

We learned from the coronavirus (COVID-19) pandemic that we can focus our attention on the healthcare system with a clear purpose when there is an imminent threat to human health. Planetary health should be no exception, especially when there is an intricate link between those two. There is a growing recognition of the impacts of climate change on health and healthcare.^3,4^ It is known that the health sector is a significant contributor to the Greenhouse Gas (GHG) emissions. The Canadian health sector is among the top four most carbon-intensive healthcare polluters on the planet, resulting in 4.6% of Canada’s total GHG emissions (nearly on a par with the country’s aviation sector).^5^ Climate change affects the social and environmental determinants of health (e.g. clean air, safe drinking water, sufficient food, and secure shelter).^3^ And so, the effects of climate change have a disproportionally negative impact on the health of communities, particularly the racialized, marginalized, and low-income populations.^6^

With the growing number of severe weather events and associated disasters, healthcare institutions are starting to consider an evolving array of strategies for enhancing resilience of the system, while also focusing on lessening of the negative impacts.^7^ It is understood that, while not all health leaders view climate change through the same lens of cause-and-effect, they are nevertheless starting to consider the need for taking action to mitigate the environmental impact of their practice, strengthen their institution’s infrastructure, and thus enhance their system’s resilience (i.e. the ability to quickly adapt and recover from changes to the environment through implementation of risk management, contingency, and continuity planning).^7^

Accreditation standards for governing boards now require leaders to consider environmental stewardship in their strategic plans.^8^ For example, the recent standards developed by the Health Standards Organization and implemented by Accreditation Canada, although not expecting hospitals to integrate environmental stewardship into their strategic plans yet, they do require governing boards to ensure that the organization promotes environmental stewardship in their operations.^8^ For this, boards also need to understand and be aware of the links between climate change and health.

This can affect many enterprises in healthcare, including procurement policies and practices. We believe that healthcare governing boards, executives, medical professionals, and other staff members should all play a role that must extend beyond the boundaries of their own institutions. Actions can influence medical professionals, patients and their families, healthcare suppliers, and entire communities. Thus, leaders of anchor institutions (i.e. hospitals, universities, and organizations that play an essential role in their local communities) can play a vital role in leading by example.

## Enhancing mitigation and adaptation to climate change: Lessons learned

The authors of this article are from Partnerships for Environmental Action by Clinicians and Communities for Healthcare Facilities (PEACH) Health Ontario, a network of clinicians and administrators who have been working together to make the healthcare system more environmentally sustainable (www.peachhealthontario.com). The PEACH team has tried to shine a light on the interventions in hospital settings that have the most impact on GHG reductions, and which may be least costly to implement, as further highlighted below.

### High impact interventions

Our work focused on seven different categories of resource utilization within hospitals (leadership, education, buildings and energy, pharmaceuticals and devices, supply chain, food, and transportation), which led to identifying tangible examples of both common interventions and lower cost/higher impact interventions.^9^ The PEACH team in partnership with the Canadian Coalition for Green Health Care has created a user-friendly, visual knowledge translation tool applicable to hospital settings, which could be used by clinicians and/or administrators to compare the environmental impact of various interventions in order to prioritize sustainability initiatives within their own healthcare practice.^10^ It is the hope this work may add to the multitude of other initiatives to help pave a way forward for curbing the carbon footprint of hospitals.^9^

### Medication overprescribing

Among all interventions we studied, reducing medication overprescribing by physicians appears to be an important carbon reduction target and perhaps among those most urgent to execute. Evidence from the top polluting countries, including Canada, is still limited, but recent data from the United Kingdom estimated that at least 10% of the total number of medications in primary care are unnecessary.^11^ By extrapolation, encouraging the medication review and optimization upon hospital admission in Canada may promote more sustainable prescribing while minimizing environmental impacts and harms. In Canada, 25% of the healthcare GHG emissions are generated by pharmaceuticals.^5^ We believe that an appropriate prescribing campaign to reduce medication overuse by 5% in a Canadian medium-sized hospital setting could result in a yearly carbon emission reduction over 100 tonnes, while decreasing pharmacy expenses significantly.^10^ However, it is still challenging to set carbon targets for the reduction of overprescribing, mostly because of the limited research to date. The savings accumulated by a more judicious use of hospital resources could be applied to other aspects of care, including reinvesting the money into other carbon reducing initiatives or purchasing better technology to enhance care.

### Supply chain impact

The supply chains within healthcare appear to generate the majority of GHGs in the system.^5^ However, it is still difficult to perform an accurate product life cycle analysis, which is a systematic analysis of the environmental impact of a product associated with all stages of their entire life cycle, of every individual item which we use. The current situation is that hospitals and procurement agencies still do not award contracts for items based on environmental sustainability of the supplier. Once environmental sustainability is added to the weighting of these contracts, we believe it will be a potential turning point in the healthcare industry. While the potential for GHG mitigation here could be significant, we can further cogitate about the actual impact.

### Culture of sustainability

In order to reach carbon net-zero emissions, healthcare institutions will need to develop a culture of sustainability, which combines both top-down and bottom-up management approaches. Such initiatives and changes can “make business sense” and help with financial sustainability. The savings accumulated by responsible use of resources could be reinvested into other hospital operations to allow for more patients to be taken care of without additional investment from taxpayers and the government. Emphasizing high impact greening initiatives that can be started immediately, while gradually creating a new corporate culture, should be encouraged. Having said that, the healthcare system is currently still facing the two global emergencies (the climate crisis and the pandemic consequences). In the race against time to cut emissions, we are concerned that the increased clinical demands and significant staffing shortages currently experienced in many workplaces may delay the process of creating a culture of sustainability. Notwithstanding all vicissitudes, we must forge ahead.

Moreover, it is known that the health effects of climate change have a disproportionally negative impact on patients from minority groups and low-income populations.^5^ Dialogues on improving sustainability of healthcare without addressing health equity would be a hollow victory. This will be in keeping with achieving the Quintuple Aim, which focuses not only on the pursuit of better health, improved patient outcomes, lower healthcare costs, and clinician wellbeing but also on health equity, and is an investment that has the potential to be a game-changer, not just for society but for the economy as well.^12^ Developing a culture of sustainable procurement should be based on the principle that environmental, ethical, and social factors are all considered in decision-making and long-term financial profitability.

## The way forward: What can be done

To build a safer, low-carbon healthcare system, we herein outline three key steps with actions that leaders could initiate and provide support for their institutions in order to move toward a more climate-resilient, carbon net-zero, and environmentally sustainable health system—through making new standards, meeting these standards, and going beyond. These ideas discussed below represent a summary of initiatives which have been well detailed elsewhere, and the reader is directed to learn more about these enterprises at the PEACH Health Ontario’s web site.^9^

### Step 1: Take immediate high impact action

Decreasing the GHG emissions must start happening now. Healthcare institutions may be at different stage and pace, from just starting their climate action journey, to transitioning to a greener system, to looking for new ideas to implement within an already robust sustainable system.

Achieving a rapid global decarbonization to stabilize the climate critically depends on galvanizing fast-spreading processes within the next few years.^1^ As such, we believe that all sectors need to go after the highest impact GHG items first. Leadership must have a strategy and assign a most responsible person.

Leaders and clinicians must look at immediate opportunities to decrease use of the biggest GHG emission items. Remarkably, up to 30% of tests, procedures, and treatments are potentially unnecessary in Canada.^13^ For example, 23% of inpatients with delirium had a potentially unnecessary CT scan of head; one in ten seniors uses a benzodiazepine on a regular basis despite this not being recommended by experts; and youth and young adults have experienced increased rates of low-dose quetiapine use, commonly used to treat insomnia, even though this is not recommended by experts.^13^ Furthermore, picking items that have cost savings (e.g. deprescribing, strategies from *Choosing Wisely* and other stewardship programs, hiring an energy manager) and then reinvesting into more costly items may seem plausible.

The Canadian Medical Association has recently partnered with *The Lancet* to highlight targeted recommendations to address the implications of climate change on human health in Canada, including communicating evidence-based solutions.^14^ Based on this 2022 report, we know that emissions from the supply chain, other than energy, is the biggest problem. This might be as high as 90% of healthcare’s GHG emissions based on the newest studies.^14^ Decisions made now will lock us into our future net-zero pathways (e.g. facilitating environmentally friendly contracts for personal protective equipment and pharmaceutical procurement, installing new boilers).

Medications are the largest single item within the supply chain with respect to carbon footprint. We need to implement safe and judicious deprescribing, especially in older aged patients where polypharmacy is prevalent.^15^ Moreover, in Sweden, medications are graded on their environmental effects, and clinicians are required to prescribe a less environmentally damaging drug where the option exists.^16,17^ It is worth considering generalizability of such initiative to apply to other populations.

Investing in fossil fuels is also a problem, and foundations need to start investing primarily in green energy. The influence that healthcare can have in reducing emissions by others is significant. There is an imperative to adapt to climate change (for the present) and build resilience (for the future).

### Step 2: Go within the facility

In order for any organization to reach net-zero carbon, they will need to look at every corner and every facet of their operations. Environmental sustainability needs to become part of the culture of the hospital, just like safety. Leaders need to communicate transparently with the healthcare workers. They need to encourage the climate passionate employees to lead groups. There are things that happen within every unit of a hospital, which only the workers in that unit can understand. They are the ones that need to think about how to make their operation run in the more sustainable way and bring these ideas forward to management.

Leaders and clinicians alike also need to encourage education about sustainability. Employers must recognize that employees want to work in a green facility. Creating transparency and accountability around employee wellbeing and wellness is no longer a “nice-to-have” initiative, but a rather vital part of doing business in the contemporary world, because leaders now may understand that the wellness of their employees could potentially have a direct impact on organizational performance.^18,19^ Furthermore, taking action on climate change in order to become a more sustainable business operation can become a wellness initiative for employees.^20,21^ Giving healthcare workers some autonomy toward making their workplace something to be proud of can be strategic.

The healthcare system itself should make a creditable commitment to environmental stewardship. Creating a culture of environmental stewardship may take years; however, engagement of frontline healthcare workers and leaders through education, collaboration, and peer support can be a call to action in the short term. Setting the tone by adopting a net-zero strategy to work toward, establishing targets, measuring results, and appointing individuals who can develop policies and procedures, can affirm that in your organization, environmental stewardship really matters.

### Step 3: Go beyond the facility and into the community

Hospitals are anchor institutions rooted by place. As a result, health leaders need to lead sustainability in our communities. Not only do hospitals need to reach net-zero carbon, but they also need to let the community know how important this is to them. Hospitals also need to celebrate their successes in the community.

Hospitals are not stand alone entities and need to lead on the sustainable procurement front. Hospitals share many of the same suppliers as other sectors including universities and municipalities. We all buy computers and office furniture, and we all need to be sending the same message that products must be sustainable and part of a circular economy (i.e. a model of production and consumption that keeps materials, products, and services in circulation for as long possible).^22^ Hospitals can also help the surrounding community by designing their natural spaces to be resilient to climate disaster events including storms and flooding.

There is also an opportunity for healthcare organizations to have a collective impact on environmental stewardship. Leaders could position hospitals as integrated partners, who have the capacity and expertise in supply chain management, to provide leadership to integrated back office procurement amongst community’s healthcare providers to further reduce the impact of supply chain on GHG production.

## Express checkout points: Reflection on the uptake of climate change mitigation

Table 1 summarizes relevant initiatives comprised of items from the three steps described previously for promoting and implementing a culture of sustainability and climate action in healthcare through work, the workforce, and the workplace.^9^

**Table 1.** Proposed strategic actions for leaders to promote a culture of sustainability and climate action in healthcare^9^.

Pick items that have cost savings (e.g. appropriate prescribing, implementing *Choosing Wisely* strategies, hiring an energy manager); then re-invest into more costly items
Change procurement contracts to include weighting for sustainability
Divest funds in fossil fuels and invest in green energy and green technologies
Designate a diverse leadership team responsible for promoting engagement (climate inaction can demoralize staff, leading to decreased efficiency)
Foster a culture of sustainability; make sustainability an item on every agenda; promote/support local green teams; encourage education about sustainability; link sustainability initiatives to employee wellness
Foster partnership in integrated healthcare systems between hospitals and community healthcare organizations as it relates to sustainable procurement
Communicate actively with the community regarding why meeting the net-zero carbon target is important to all (hospitals are anchor institutions which need to lead sustainability)

As a final reflection for the reader, think about the following when considering climate action items at your healthcare facility:

— What do you think your institution needs in order to address this climate action?
— Who do you need to collaborate with, both inside and outside your institution, to make it happen?
— What impact can you expect to have on your patients as a healthcare provider, your medical professional colleagues, community, stakeholders, and the bottom line?
— How can you encourage learning, creativity, innovation, and engagement in climate action initiatives at both your workplace and in your community?
